# Strong light scattering and broadband (UV to IR) photoabsorption in stretchable 3D hybrid architectures based on Aerographite decorated by ZnO nanocrystallites

**DOI:** 10.1038/srep32913

**Published:** 2016-09-12

**Authors:** Ion Tiginyanu, Lidia Ghimpu, Jorit Gröttrup, Vitalie Postolache, Matthias Mecklenburg, Marion A. Stevens-Kalceff, Veaceslav Ursaki, Nader Payami, Robert Feidenhansl, Karl Schulte, Rainer Adelung, Yogendra Kumar Mishra

**Affiliations:** 1 National Center for Materials Study and Testing, Technical University of Moldova, Block 1, Bulevardul Stefan cel Mare si Sfînt 168, Chisinău 2004, Moldova; 2 Institute of Electronic Engineering and Nanotechnologies, Academy of Sciences of Moldova, Stefan cel Mare av. 1, MD-2001 Chisinau, Republic of Moldova; 3 Functional Nanomaterials, Institute for Materials Science, Kiel University, Kaiserstr. 2, D-24143 Kiel, Germany; 4 Institute of Polymers and Composites, Hamburg University of Technology, Denickestr. 15, D-21073 Hamburg, Germany; 5 School of Physics, University of New South Wales, NSW 2052 Sydney, Australia; 6 Niels Bohr Institute, University of Copenhagen, Universitetsparken 5, DK-2100 Copenhagen, Denmark

## Abstract

In present work, the nano- and microscale tetrapods from zinc oxide were integrated on the surface of Aerographite material (as backbone) in carbon-metal oxide hybrid hierarchical network via a simple and single step magnetron sputtering process. The fabricated hybrid networks are characterized for morphology, microstructural and optical properties. The cathodoluminescence investigations revealed interesting luminescence features related to carbon impurities and inherent host defects in zinc oxide. Because of the wide bandgap of zinc oxide and its intrinsic defects, the hybrid network absorbs light in the UV and visible regions, however, this broadband photoabsorption behavior extends to the infrared (IR) region due to the dependence of the optical properties of ZnO architectures upon size and shape of constituent nanostructures and their doping by carbon impurities. Such a phenomenon of broadband photoabsorption ranging from UV to IR for zinc oxide based hybrid materials is novel. Additionally, the fabricated network exhibits strong visible light scattering behavior. The developed Aerographite/nanocrystalline ZnO hybrid network materials, equipped with broadband photoabsorption and strong light scattering, are very promising candidates for optoelectronic technologies.

Hybrid nanomaterials in the form of combination of several components equipped with different individual nanoscale features are very important material candidates from an application point of view because, in hybrid form, most of the desired properties are accumulated together, often resulting in the occurrence of entirely new characteristics^1^,^2^,^3^,^4^. Due to multifunctional properties, hybrid materials are leading the trends in materials synthesis and application communities^1^,^3^,^4^,^5^,^6^,^7^. Looking on their future technological potentials, many new strategies for fabricating various hybrid nanomaterials are being introduced and accordingly investigations are continuing, but this field still has to come up with further cost-effective approaches which can offer simple and mass-scale fabrication of desired materials in appropriate hybrid forms. At the same time, it is also important to overcome the utilization complexities related to the requirement of nanostructure’s integration on the micro-chip, e.g., focused ion beam, lithography, etc., have been mainly used to develop electronic devices based on individual nanowires^8^,^9^,^10^,^11^,^12^,^13^. To efficiently utilize the excellent nanoscale features, three-dimensional (3D) forms of nanomaterials are the most appropriate candidates and currently significant efforts are being undertaken for the purpose of fabricating different types of 3D nanomaterials, from inorganic components^14^,^15^,^16^,^17^,^18^, carbon/graphene^8^,^9^,^10^,^11^,^12^,^19^,^20^, and others along with their wide range of applications. The appropriate combination of inorganic and carbon materials in 3D architectures is very attractive, because, in this form, the important properties of all the nanoscale counterparts become easily accessible^8^,^9^,^10^,^11^,^12^,^19^,^20^. Although 3D nanomaterials, in the form of porous interconnected networks built from nanoscopic building blocks, are very relevant with respect to different technological applications, their cost-effective fabrication using simple processing steps is still an open issue, normally it requires complicated bottom-up or top-down strategies^21^,^22^. In contrast to conventional lithography techniques, recently a new approach based on flame synthesis has been introduced and it offers very simple and mass scale fabrication of highly porous 3D interconnected ceramic zinc oxide (ZnO) networks^23^,^24^. This flame based strategy involves direct synthesis of zinc oxide nano- and microscale tetrapods from metallic zinc microparticles in flame via solid-vapor-solid growth process^25^ and these tetrapod structures have already demonstrated their application potentials in various advanced technologies^26^,^27^,^28^,^29^,^30^,^31^.

From metal oxides family, ZnO is one of the most studied semiconductor compounds, and the number of publications devoted to this material continues to increase with time, especially those related to low-dimensional structures such as nanodots, nanorods, nanobelts, nanotubes, nanotetrapods, nano-multipods etc. Along with the industrial applications related to piezoelectric properties of zinc oxide, nanocrystalline ZnO represents a valuable perspective material for a variety of other applications^32^,^33^. An important emerging practical use of ZnO nanomaterial is related to biomedical applications, e.g., bioimaging, biosensing, drug delivery, gene delivery, antimicrobial effect, stimulation or inhibition of biological processes in living systems, from single cells to humans^34^,^35^. Over the recent years, various approaches have been applied for the purpose of building hierarchical three-dimensional nano-ZnO architectures with enhanced photocatalytic, photoelectric and gas-sensing performances, e.g., soft-chemical approach^36^,^37^, hydrothermal synthesis^18^,^38^,^39^, electrodeposition^17^, chemical vapour deposition^40^, thermal evaporation^41^,^42^, etc. In particular, Liu *et al*.^42^ thermally deposited the zinc oxide on bundles of the graphite fibers with the average diameter of 7 μm. As a result, the formation of graphite fiber/ZnO core-shell structures was realized, the ZnO shell consisting of microtubes was covered by rods with ~400 nm diameter and ~5-μm length. Later, these researchers succeeded in fabricating hollow ZnO microtube-nanorod structures by oxidizing the central graphitic fiber in air at 800 °C for 20 min^42^. In contrast to above mentioned synthesis process, the present flame based approach offers a simple 3D nanostructuring using ZnO tetrapods and these networks can be further functionalized with other metal oxides for multifunctional applications^23^,^24^ including fabrication of new varieties of 3D networks, for example, the carbon based highly porous 3D Aerographite (AG) material^43^ and its other variants in form of GaN-Aerographite hybrid networks^44^.

Stretchable materials equipped with interesting functionalities from nanoscale inorganic materials are becoming more and more important because of their interesting applications, for example, in bendable sensing devices and artificial skin^45^, etc., and they can be easily fabricated by using appropriate templates with desired porosity and flexibilities. Being very light in weight, extremely porous (>99.95%), and mechanically stable enough, these carbon based Aerographite networks could be used to fabricate smart 3D nanomaterials where functionalities and stretchability are simultaneously desired, e.g., in flexible 3D luminescent elements. Additionally, it is necessary to emphasize here, that even if the entire surface of Aerographite network is coated with inorganic nano- and microstructures, for example, GaN nanocrystals, etc., the resulting hybrid networks still retain a high degree of flexibility^44^ which is of paramount importance for many applications. Very recently, advanced scattering elements have received significant attention due to the upcoming developments in modern lighting technologies based on light emitting devices and laser diodes. If the Aerographite networks can be decorated with appropriate inorganic nanostructures having very high surface area in hybrid 3D form, the light scattering behaviour can then be simply tuned by compressing/expanding the hybrid network which could be useful for large range of optoelectronic or photodetector applications. Accordingly, here we report on the formation of hierarchical 3D Aerographite/nanocrystalline ZnO (AG/ZnO) architectures as a result of zinc oxide deposition on flexible Aerographite scaffolds by using RF magnetron sputtering techniques, and demonstrate that this hybrid 3D nanomaterial exhibits significant technological perspectives in terms of optical properties such as light emission, broadband photo-detection, and light scattering behaviour.

## Results and Discussion

**Figure 1:**
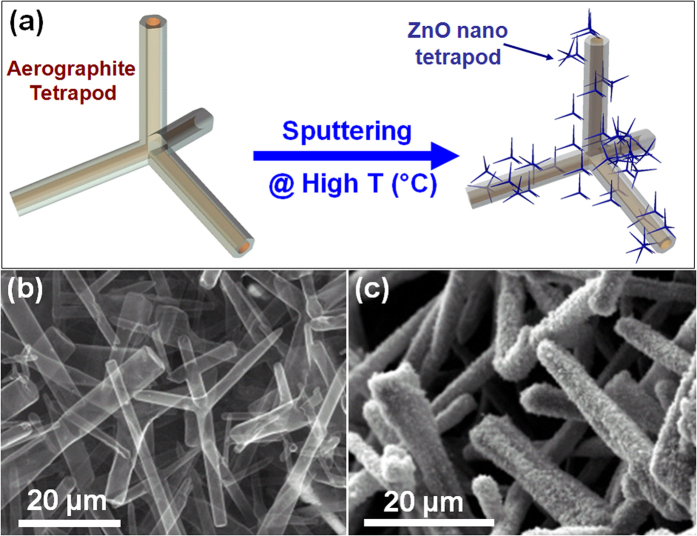
Aerographite (AG) nano- and microtubular hollow tetrapods (AGHT) as universal templates for growing hybrid 3D nanomaterials: (**a**) Fabrication concept for decorating the AGHT with ZnO nanotetrapods (ZnO-nT) using a single step sputtering process. By changing the deposition time, the morphology of loaded nanostructures can be tailored. (**b**–**c**) Typical SEM images demonstrating the morphologies of AGHT backbone before and after loading with ZnO nanostructures respectively.

Figure 1(a) illustrates schematically an individual Aerographite micro-tetrapod before and after deposition of ZnO nanoscale tetrapods, while Fig. 1(b,c) shows the morphology of the as-grown Aerographite and of the Aerographite/ZnO hybrid nanomaterial. Aerographite represents an ultra-light weight, extremely porous, mechanically flexible, graphite based 3D network built from interconnected struts of hollow graphite tubes with micrometer-scale diameters and a nanoscopic (~15 nm) wall thickness^43^. The previous investigations^44^ carried out on Aerographite-GaN hybrid networks demonstrated that the Aerographite scaffold can sustain high loading densities of semiconductor nano-and microcrystallites on its huge specific surface without visible structural deformations.

**Figure 2:**
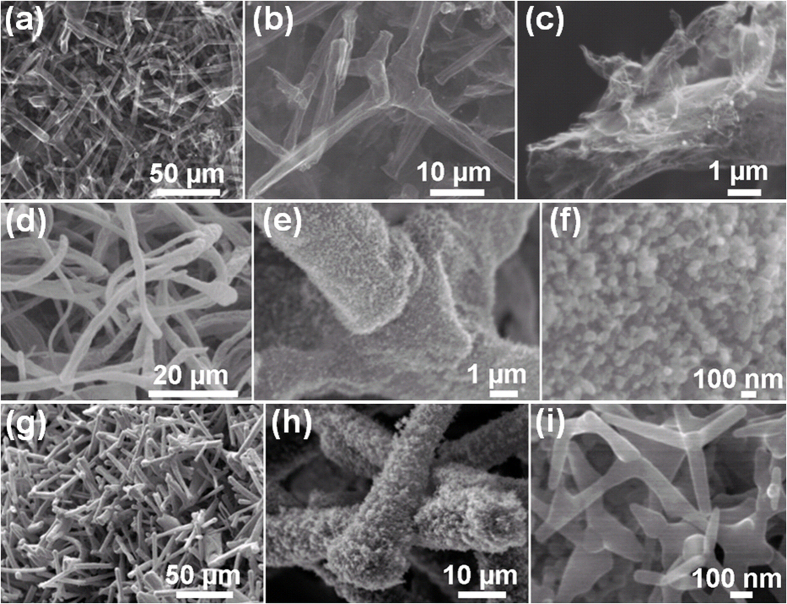
SEM images demonstrating the morphologies of AGHT networks loaded with different types of ZnO nanostructures. (**a**–**c**) SEM images at increasing magnifications (left to right corresponding to AG templates decorated with well separated ZnO nanodots. (**d**–**f**) Longer deposition time leads to complete coverage of AG tubes with ZnO nanocrystals as can be seen in zoomed series of SEM images (left to right). (**g**–**i**) Under appropriate deposition conditions, growth of ZnO nanotetrapods occurs which leads to the formation of hybrid material in form of self-assembled hierarchical networks of ZnO nanotetrapods on the AGHT architectures, the SEM morphologies at increasing magnifications (left to right) demonstrate such a hybrid network. The arm thickness of ZnO nT is ~100 nm.

The evolution of the morphology and micro-structure of the deposited ZnO nanostructures with respect to the duration of RF magnetron sputtering was studied by scanning electron microscopy (SEM) and the corresponding results are illustrated in Fig. 2(a–i). A 10 nm thick film deposition at 90 °C with subsequent heat treatment of the specimens at 490 °C results in the formation of nanoscopic crystallites from ZnO which are clearly seen in high resolution SEM image, see Fig. 2(c). With increase of the deposition time, the outer surface of the Aerographite microtubes in the network gets homogenously covered with a continuous film of nanocrystalline ZnO, see Fig. 2(d–f). It is evident from Fig. 2(f), that the deposited 150 nm thick ZnO film exhibits a nano-granular structure with the average nanocrystal size of about 100 nm.

Deposition of films with the thickness greater than or equal to 1 μm is accompanied by considerable changes in the morphology and micro-structure of the deposited ZnO layers (Fig. 2(g–i)). In such cases the outer part of the deposited ZnO film appears to be highly porous, mainly due to the formation of sub-micrometer-scale ZnO tetrapods, see the high-resolution image in Fig. 2(i). Note that the diameter of the tetrapod arms is of ~100 nm or less. Interestingly, long duration ZnO deposition with subsequent heat treatment of specimens at 490 °C leads to the formation of micron-scale tetrapods weakly bound to the Aerographite/ZnO hybrid architecture which leads to the possibility of detaching the ZnO nano- and micro-tetrapods and gathering them on a foreign substrate by simply shaking up the as-prepared AG/ZnO network (Supplementary Information, Figure S1). The tetrapod growth is initiated by the emergence of a nucleus with subsequent formation of four arms and the nature of nuclei depends upon the involved synthesis process. The growth mechanism of ZnO nano- and microscale tetrapods by different synthesis processes is already a long discussed topic in the literature^25^,^46^,^47^,^48^. The present results pave the way for the controlled fabrication of micrometer-scale ZnO tetrapods with sub 100 nm thickness of the constituent arms. It is worth mentioning in this regard, that the dimensions of the initial ZnO tetrapods/multipods used to fabricate the Aerographite network were of several tens of micrometers, the transverse dimensions of the constituent arms being of micrometer scale^43^. As in the case of GaN deposition^44^, the Aerographite network sustains a high amount of loading of nanocrystalline ZnO, in spite of the relatively thin nanoscopic walls (~15 nm thick) of the constituent graphitic microtubular architecture. In contrast to previously reported deposition of GaN on both the inner and outer surface of AG tubes^44^, the present results show that the ZnO deposition takes place only on the outer surface of graphitic microtubular structures.

The crystallinity of the ZnO deposited on the Aerographite scaffold was studied by micro-Raman scattering techniques. Note that the Raman spectrum of bulk wurtzite-type ZnO is characterized by the following Raman-active phonon modes: A_1_(TO) at 379 cm^−1^; E_1_(TO) at 410 cm^−1^; E_2_(high) at 439 cm^−1^ and E_1_(LO) at 591 cm^−1 ^49. According to the micro-Raman results (see Figure S2, Supplementary Information), the vibrational modes mentioned above are inherent also to nanocrystalline ZnO deposited on the Aerographite network. The redshift of the LO peak in nanocrystalline ZnO in comparison with bulk ZnO can be attributed to local heating induced by the focused laser beam from Raman instrument. Local heating is also responsible for the observed red shift of the E_2_(high) mode in nanocrystalline ZnO (438.1 cm^−1^) in comparison to that of bulk ZnO. The two additional Raman peaks at low-frequencies of ~278 and 331 cm^−1^ are most likely attributed to the second order Raman processes involving acoustic phonons^50^,^51^. The deposited ZnO nanostructures in the present AG/ZnO hybrid network basically exhibit tetrapod morphology and it is important to mention here that previous studies, including those discussing the models of tetrapod growth, have demonstrated the wurtzite crystal structure of ZnO nanorods constituting the arms of ZnO tetrapods^52^. At the same time, the wurtzite structure of ZnO nanorods has also been confirmed by detailed X-ray diffraction, high resolution transmission electron microscopy and selected area electron diffraction studies^25^.

**Figure 3:**
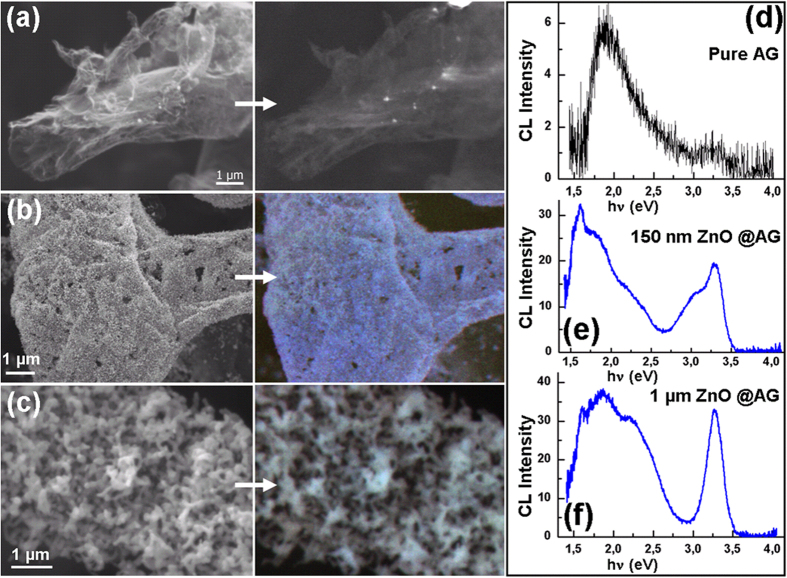
Cathodoluminescence studies on ZnO-Aerographite hybrid networks. With increase in deposition time, the growth behavior of ZnO structures varies from well separated ZnO nanodots to self-assembled hierarchal network of ZnO nanotetrapods. (**a**–**c**) SEM images for AG networks coated with ZnO at different deposition times (low to high) and corresponding μ-CL images (marked with arrow on right, see text for details). The CL spectra corresponding to Aerographite coated by ZnO nanodots, 150 nm ZnO @ AG, and 1 μm ZnO @ AG are shown in (**d**–**f**) respectively. The increase in intensity of UV exciton band emission peak for 1 μm ZnO film (**f**) is clear indication for the growth of crystalline ZnO nanostructures. The feature at ~1.6 eV in (**e**,**f**) is a second order grating artefact.

Taking into account that ZnO is an important optoelectronic material, detailed cathodoluminescence investigations on AG/ZnO 3D architectures have been performed. Figure 3(a) illustrates the SEM image and panchromatic CL image taken from a fragment of AG/ZnO network after deposition of 10 nm thick layer at 90 °C with subsequent heat treatment of the specimen at 490 °C, while Fig. 3(b,c) show the SEM images and the colour-composite (360 + 400 + 525) nm CL images taken from AG/ZnO hybrids with the thickness of the deposited ZnO layer of ~150 nm and ~1 μm, respectively. The analysis of CL spectra demonstrated in Fig. 3(d–f) is indicative of several stages of the formation of ZnO nano/micro-structures on the Aerographite scaffold. In the very first stage, the ZnO nanodots are formed at the interface, as shown in Fig. 3a. These nanodots as well as the interfacial layer are highly doped with carbon. The microstructural analysis suggests that the interfacial layer comprises ZnO and Carbon in form of a two phase nanocomposite material. A very weak and broad CL band is emitted from ZnO nanodots and interfacial layer in the visible range, as shown in Fig. 3d. With increasing the thickness of the ZnO granulated layer, the level of doping with carbon decreases and emission in the UV region emerges in the spectrum, as observed in Fig. 3e. The ratio of the UV to visible emission increases in the upper ZnO layer formed of nanotetrapods. This indicates the decrease in the level of doping with increasing distance from the Aerographite/ZnO interface, which can be attributed to the limited diffusion of carbon.

The analysis of the CL spectrum in Fig. 3e shows that the UV and violet emission consists of two bands located at ~3.28 eV and ~3.1 eV, while the emission in the visible range is composed of at least two bands with maxima around 1.8–1.9 eV and 2.2–2.3 eV, respectively. With increasing the thickness of the deposited ZnO layer, the emission band at 3.1 eV vanishes, while the relative intensity of the red band at 1.8–1.9 eV decreases substantially (Fig. 3f). It has been previously observed that the UV band at ~3.28 eV represents a superposition of the zero-phonon line and the phonon replicas of the free exciton emission^41^. The presence of the free exciton emission is indicative of the high crystalline quality of the ZnO nanotetrapods. As far as the emission band at 3.1 eV is concerned, the luminescence in this region was previously suggested to appear from recombinations involving the Zn interstitials (Zn_i_) with the maximum at ~2.9 eV, or Zn vacancies (V_Zn_) with the maximum at ~3.06 eV^53^. In addition, a blue and violet luminescence band around ~3.0 eV has been observed in carbon modified ZnO nanoparticles, and this emission is attributed to the carbon adsorbed at the O^2−^ vacancies existing in large quantities at the ZnO nanoparticles surfaces, therefore creating carbon substitution of oxygen (C_O_)^54^. It has been suggested that the blue/violet emission may originate from the recombination of the electron in the conduction band or in a shallow donor with the C_O_ acceptor. The carbon enhanced blue/violet luminescence at ~3 eV has also been observed in ZnO films grown by pulsed laser deposition techniques^55^. Note that similar blue/violet emission was reported for GaN doped with carbon which was assigned to C_N_ acceptor^56^.

On the basis of the literature reports^57^,^58^,^59^,^60^,^61^, the luminescence band at ~2.9 eV is most likely due to Zn interstitials and the band at ~3.06 eV is associated to Zn vacancies. It has been concluded that the carbon impurities promote the zinc related native defects in ZnO and may also generate radiative recombination channels due to the carbon impurity incorporated in the ZnO lattice. Therefore, the following defects can be considered as the origin of the blue/violet emission from ZnO nanoparticles: Zn_i_, V_Zn_, and C_O_. However, the Zn interstitials have high formation energies in *n*-type ZnO^57^. In addition, these defects are unstable at room temperature^58^,^59^, and are easily annealed even below room temperature (at ~170 K)^60^. According to the McCluskey *et al*.^59^, the Zn vacancies are most probably responsible for the observed green emission at ~2.35 eV^61^, rather than for the blue/violet emission. The absence of the CL band at ~3.1 eV in the less doped nanoscale ZnO tetrapods in comparison with the highly carbon doped ZnO nanogranular layer closer to the Aerographite/ZnO interface, corroborates the suggestion that C_O_ defects are a source of the blue/violet emission in this AG/ZnO hybrid nanomaterial.

**Figure 4:**
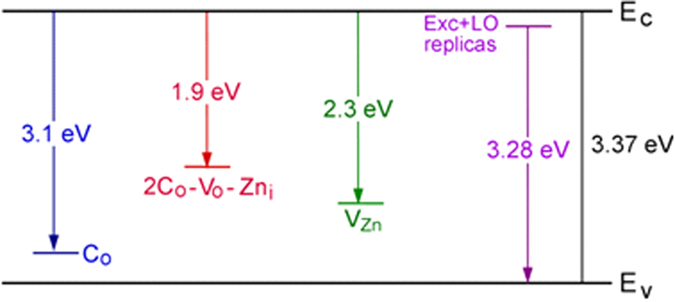
The scheme of defect levels responsible for CL bands.

The CL band at ~1.9 eV also seems to be related to the carbon impurity, in the form of a complex defect 2C_O_-V_O_-Zn_i_ with an energy level situated 1.9 eV below the conduction band of ZnO, according to the theoretical calculations by Lu *et al*.^62^. The green emission at ~2.3 eV does not seem to be influenced by carbon doping in AG/ZnO hybrid samples, since the ratio of its intensity to the intensity of the excitonic emission at ~3.28 eV is practically the same in ZnO nanotetrapods and in ZnO nanograins (comparing Fig. 3e,f). In this respect, the emission at ~2.3 eV can be attributed to the intrinsic defects, most probably to zinc vacancies as described above. Although it was believed in previous investigations that the green emission in ZnO is associated with Cu impurities^63^,^64^,^65^,^66^, oxygen vacancies^67^,^68^,^69^,^70^,^71^,^72^,^73^, or oxygen antisites^53^,^74^, the analysis carried out later by McCluskey *et al*., demonstrated that the green PL is not due to O vacancies^59^. The broad CL absorption band corresponding to Fig. 3f was deconvoluted into several peaks (shown in Figure S3, Supplementary Information) and they are in good agreement with the mentioned defect levels within the ZnO bandgap. It is worth noting here that defects such as O antisites, Zn antisites, and O interstitials have high formation energies, and therefore are probably not present in significant concentrations under normal conditions^59^. Figure 4 presents a scheme of defect levels responsible for CL bands observed in AG/ZnO hybrid nanomaterials.

**Figure 5:**
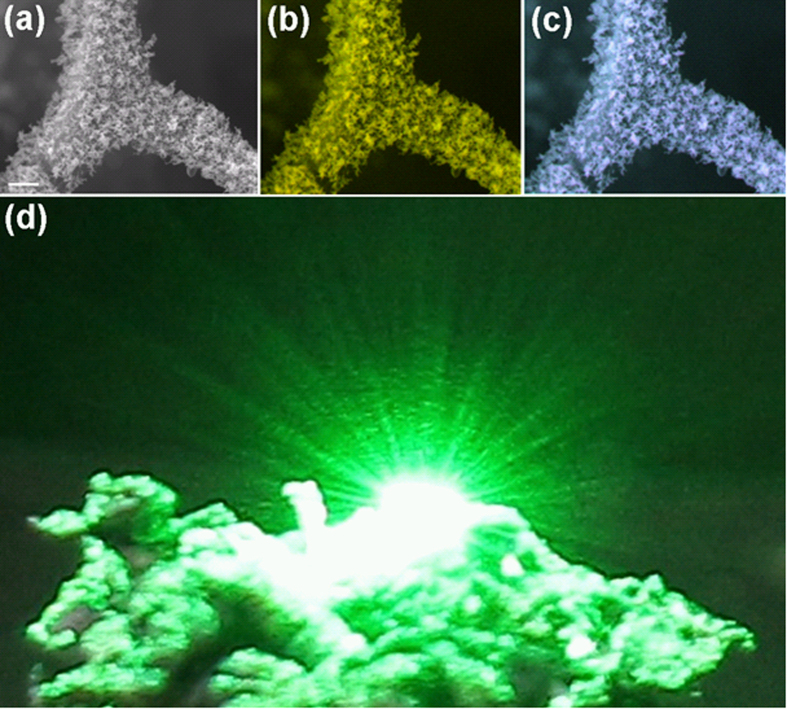
(**a**) SEM image of a fragment of Aerographite covered by ZnO tetrapods (scale bar 1 μm), and color-composite μ-CL images: (525 + 675) nm (**b**), and (360 + 400 + 525) nm (**c**). (**d**) Illustrates the scattering behaviour of green light generated by a laser pointer.

Deposition of relatively thick nanostructured ZnO layers on Aerographite tubular networks results in the formation of hybrid nanomaterial with enhanced optical properties. Figure 5(a) presents a fragment of the Aerographite tetrapod-like structure covered by ZnO nano- and microtetrapods, while Fig. 5(b,c) show color-composite μ-CL images: (525 + 675) nm in (b), and (360 + 400 + 525) nm in (c). It has been found that the Aerographite/ZnO hybrid sample exhibits strong scattering of electromagnetic radiation in the whole visible region. Figure 5(d) illustrates the scattering of green light generated by a laser pointer. The reason for such strong scattering is obviously the correlation of the light wavelength with the dimensions of the deposited ZnO nano- and microtetrapods. Aerographite network does not exhibit any light scattering behavior (it is perfectly black and everything gets absorbed on it) and hence the observed large scattering of green light is mainly a contribution from complex shaped ZnO nano- and microscale tetrapods with very high surface area and large number of edges deposited over Aerographite scaffolds. Note that strong scattering usually leads to enhanced optical absorption in the medium due to the increase of the optical path of the electromagnetic radiation.

**Figure 6:**
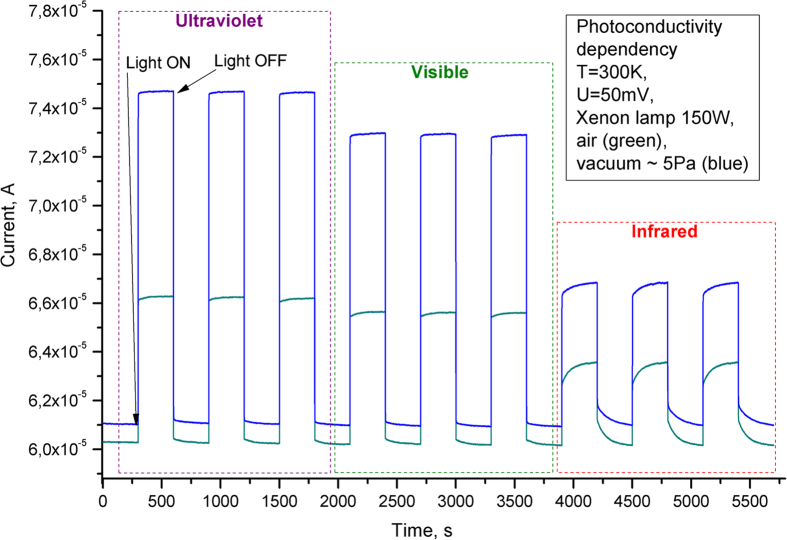
Broadband photosensitivity of the ZnO-Aerographite hybrid nanomaterial in UV, visible and infrared regions of spectrum (see text for details).

Photodetection is an important application of most of the wide bandgap metal oxide semiconductor nanostructures but they are mainly prone to exhibit current sensitivity in ultraviolet (UV) region^26^,^75^. However, it will be very helpful if the photodetection range of these materials is broadened which could be possible when they are in appropriate hybrid form. In order to investigate the photodetection, an Aerographite/ZnO hybrid network based electronic device was fabricated (device fabrication concept in Figure S4, Supplementary Information) and it’s current-voltage (I-V) characteristics (given in Figure S5, Supplementary Information) have been measured under different environmental (in dark/light, air/vacuum) conditions which reveals its strong sensitivity to surrounding environments. The resulting current switching behavior, shown in Fig. 6, demonstrates that the Aerographite/ZnO hybrid nanomaterial exhibits strong photosensitivity in a wide spectral range from the ultraviolet to infrared (UV to IR) wavelengths. ZnO nanomaterials typically exhibit photodetection behavior in the range from UV to visible region but the present AG/ZnO hybrid material shows interesting switching behavior in the infrared region too (up to around 2.5 μm) and therefore our material has the potential to be utilized as the universal photodetector device (which is not in general common for wide bandgap semiconductors based devices). It seems that different layers of the hybrid nanomaterial are responsible for observed cathodoluminescence and broadband (UV to IR) photoconductivity (PhC) behaviors. The main CL signal is emitted from the upper tetrapod layer with a higher crystalline quality, while the lower granular layers, including that at the interface with the Aerographite scaffold, are responsible for the PhC, since the granular morphology ensures the percolation pathway of current through the material. On the other hand, the huge surface-to-volume (S/V) ratio of the nanomaterial suggests the importance of surface effects and that of oxygen molecules in the photoconductivity. It is generally believed that adsorption (in the dark) and desorption (under UV illumination) of oxygen molecules govern the photoresponse of ZnO in air^76^,^77^,^78^,^79^,^80^. In the dark, free electrons of the n-type semiconductor are captured by the oxygen molecules adsorbed on the surface of ZnO nanograins: O_2_(g) + *e*^−^ → O_2_^−^(ad). In consequence of this, a depletion layer appears in the immediate vicinity of the nanograin surface, resulting in an upward band bending near the surface. When the material is characterized by large surface-to-volume ratio, the adsorption of O_2_ significantly reduces its electrical conductivity. Excitation of ZnO by UV quanta with energies greater than *E*_g_ generates electron–hole pairs. Holes migrate to the surface along the potential slope caused by the band bending and recombine with O_2_-trapped electrons, thus releasing oxygen from the surface: O_2_^−^(ad) + h^+^ → O_2_(g). The unpaired electrons are either collected at the anode or recombine with holes generated when oxygen molecules are re-adsorbed and ionized at the surface.

It is clear from Fig. 6 that the conductivity in air is lower in comparison to that measured under reduced pressure (Figure S5, Supplementary Information). Cammi *et al*.^81^, recently explained a similar decrease as being associated with the adsorption of oxygen on the ZnO surface and trapping of electrons from the conduction band^81^. The photoconductivity is also higher under reduced pressure which is an indication that the exploitation of broadband photodetectors on the basis of AG-ZnO hybrid material in various devices, for instance in flame alarm systems or high-performance liquid chromatography devices, is even more favorable under vacuum. However, one should take into account additional costs related to evacuation and encapsulation of device structures.

The fact that the photoconductivity induced in the samples investigated here is not only due to the UV irradiation, but also due to the visible and near-infrared light irradiation, can be explained by the presence of a large amount of defect-induced energy levels within the ZnO bandgap which could also form extended band-edge tails of the density of states. As discussed above, the granular layer and the interfacial layer are highly doped with carbon, which can explain the generation of electron–hole pairs by radiation with a large spectrum of wavelengths. It is necessary to mention here that the pure Aerographite network does not exhibit any current switching behavior (Figure S6, Supplementary Information) with respect to wide range of light wavelengths (UV, white LED, white halogen lamp) and therefore the observed broadband photoswitching from the hybrid AG-ZnO network based device is mainly due to the presence of ZnO nano- and microstructures. So far the photoabsorption in ZnO has been mainly explained in terms of defects but other optical effects like whispering gallery modes (WGMs), second harmonic generation (SHG), etc. could also contribute to the broad (visible to infrared) photoabsorption. The WGM mainly depends upon the size and shape of the ZnO nano- and microstructures and WGM resonances lead to additional peaks over the broad ZnO defect luminescence peak^82^,^83^. The CL spectrum of AG-ZnO hybrid material does exhibit some additional peaks over broad ZnO defect peak which could be due to WGM resonances but further investigations are required for the explicit disclosure of their origin. As to the nonlinear optical properties, they depend upon the size of ZnO nano- and microstructures, crystalline quality, surface defects, grain boundaries as well as impurities^84^,^85^, and their contribution to the observed broadband photoabsorption in present AG-ZnO hybrid network cannot be ignored. Irrespective of the deep understanding of the origins, characteristic features of the stretchable AG-ZnO hybrid network material like tunable scattering of light and broadband (UV to IR) photoabsorption are technologically very promising for advanced optoelectronic applications.

## Conclusion

In summary, our research demonstrates that Aerographite/ZnO hybrid nanomaterials with varying ZnO morphologies, i.e. from nanocrystallites to nanotetrapods on extremely porous and highly flexible Aerographite scaffolds, can be easily fabricated by controlled deposition of ZnO in a simple and cost-effective technological process. The deposition time also enables the controlled modification of light emission properties of the 3D hybrid architectures as evidenced by the detailed cathodoluminescence investigations. The difference in luminescence properties of wurtzite ZnO thin layers is discussed in detail in terms of native defects, free excitons, and carbon doping from the AG network which is also in agreement with Raman scattering analysis. The photosensitivity of the hybrid material is explained by the adsorption (in the dark) and desorption (under illumination) of oxygen molecules to/from the ZnO surface, while the broadband photoabsorption occurs mainly due to the presence of a large amount of defect levels in the material bandgap, which may also form extended band-edge tails of the density of states in the ZnO layer in close proximity to the Aerographite scaffold. The material with relatively thick nanostructured ZnO layers obtained after long duration deposition exhibits enhanced optical properties, such as strong scattering due to the correlation of the light wavelength with the dimensions of the deposited ZnO nanotetrapods, and enhanced optical absorption in the medium caused by the light trapping, i.e., increased optical path of the electromagnetic radiation. The proposed strategy provides a cost-effective method for the fabrication of nano- and micrometer scale ZnO tetrapods with sub 100 nm arm thickness which can be easily detached from the hybrid architecture, while the resultant stretchable hybrid material could be very promising for optoelectronic technologies.

## Materials and Methods

The Aerographite networks have been synthesized by the one step chemical vapor deposition (CVD) process on metal oxide porous ceramic templates as described in a previous investigation^43^. Highly porous ceramic networks with a 3D architecture, which are entirely built up from interconnected micrometer-scale thick rods, often in the shape of tetrapods and multipods, are used as sacrificial templates^23^. The as-grown Aerographite networks have been used as templates for the growth of nanocrystalline ZnO by using RF magnetron sputtering method in a high vacuum chamber equipped with turbomolecular and mechanical pumps. The Aerographite template was mounted on a rotary support, and the distance from the target to the template was 8 cm. The base pressure was about 5 × 10^−5^ Pa, and the flow rate of Ar was kept at a constant value of 60 mL/min controlled by a mass flow controller. A disc of 99.99% pure zinc served as metal target for sputtering. The template temperature measured by using a chromel-alumel thermocouple was 90 °C. The AG scaffold was rotating during sputtering to provide conditions for uniform deposition of ZnO. The deposited film thickness was controlled during sputtering by using a MTM-10/10A high resolution thickness monitor quartz microbalance. After the deposition process, the specimens were annealed at 490 °C for 60 minutes in an oxygen atmosphere with a gas flow of ∼100 ml/min.

The morphology of AG/ZnO hybrid networks were investigated using scanning electron microscopes Zeiss Ultra Plus and VEGA TESCAN TS 5130MM. The compositional analysis of AG/ZnO networks was carried out using Energy Dispersive X-ray analysis (EDX), in combination with SEM. A JEOL 7001F Field Emission SEM equipped with a Gatan XiCLone CL microanalysis system was used for comparative morphological and CL characterization. The monochromatic CL images were collected using a Peltier cooled Hamamatsu R943-02 High Sensitivity Photomultiplier Tube. The CL spectra and images were generated from typical regions of the specimen using for excitation 10 keV, 10 nA electron beam. The CL spectra have been collected with a Princeton Instruments Pixis 100 UV optimized CCD. To carry out photoelectrical measurements, a 500 nm thick SnO_2_ film was deposited on top of ZnO layer by using RF magnetron sputtering approach described above. A target of SnO_2_ with 99.99% purity was used for this purpose. The frontal electrical contact was painted by conductive silver paste, while a transparent conductive oxide (TCO) served as rear contact. The schematic structure of TCO/aerographite/ZnO/SnO_2_/Ag specimen is illustrated in Supplementary Information, Figure S4.

Electromagnetic radiation from a Xenon lamp was used for the photoconductivity (PC) excitation. Optical filters were used to cut-off radiation from different spectral ranges (ultraviolet: 300–400 nm, power density at the sample surface 17.6 mW/cm^2^; visible 400–700 nm, power density 25.5 mW/cm^2^; and infrared 700–2500 nm, power density 134 mW/cm^2^). The current through the samples was measured by means of Keithley’s Series 2400 Source Measure Unit. Since the PC decay time is long enough, a mechanical shutter was used in the PC relaxation experiments. The signal from the Source Measure Unit was introduced in an IBM computer via IEEE-488 interface for further data processing.

## Additional Information

**How to cite this article**: Tiginyanu, I. *et al*. Strong light scattering and broadband (UV to IR) photoabsorption in stretchable 3D hybrid architectures based on Aerographite decorated by ZnO nanocrystallites. *Sci. Rep.* **6**, 32913; doi: 10.1038/srep32913 (2016).

## Supplementary Material

Supplementary Information
