# Supplementary material for: Strong light scattering and broadband (UV to IR) photoabsorption in stretchable 3D hybrid architectures based on Aerographite decorated by ZnO nanocrystallites
Source: Sci Rep. 2016 Sep 12;6:32913. doi: 10.1038/srep32913 (PMC5018730; doi:10.1038/srep32913)
Supplement: Supplementary Information [file srep32913-s1.pdf]

Supplementary information for

Strong light scattering and broadband (UV to IR) photoabsorption in stretchable 3D hybrid architectures based on Aerographite decorated by ZnO nanocrystallites

Ion Tiginyanu,[1*] Lidia Ghimpu,[1] Jorit Gröttrup,[2] Vitalie Postolache,[1] Matthias Mecklenburg,[3] Marion A. Stevens-Kalceff,[4] Veaceslav Ursaki,[1] Nader Payami,[5] Robert Feidenhansl,[5] Karl Schulte,[3] Rainer Adelung,[2*] Yogendra Kumar Mishra[2*]

[1]National Center for Materials Study and Testing, Technical University of Moldova, and Institute of Electronic Engineering and Nanotechnologies, Academy of Sciences of Moldova, Stefan cel Mare av. 1, MD-2001 Chisinau, Republic of Moldova

[2]Institute for Materials Science, Kiel University, Kaiserstr. 2, D-24143 Kiel, Germany

[3]Institute of Polymers and Composites, Hamburg University of Technology, Denickestr. 15, D-21073 Hamburg, Germany

[4]School of Physics, University of New South Wales, NSW 2052 Sydney, Australia

[5]Niels Bohr Institute, University of Copenhagen, Universitetsparken 5, DK-2100 Copenhagen, Denmark

Keywords: Metal oxide - carbon hybrid nanomaterials, 3D flexible network, strong light scatterers, luminescence, broadband (UV to IR) photoabsorbers

Figure S1

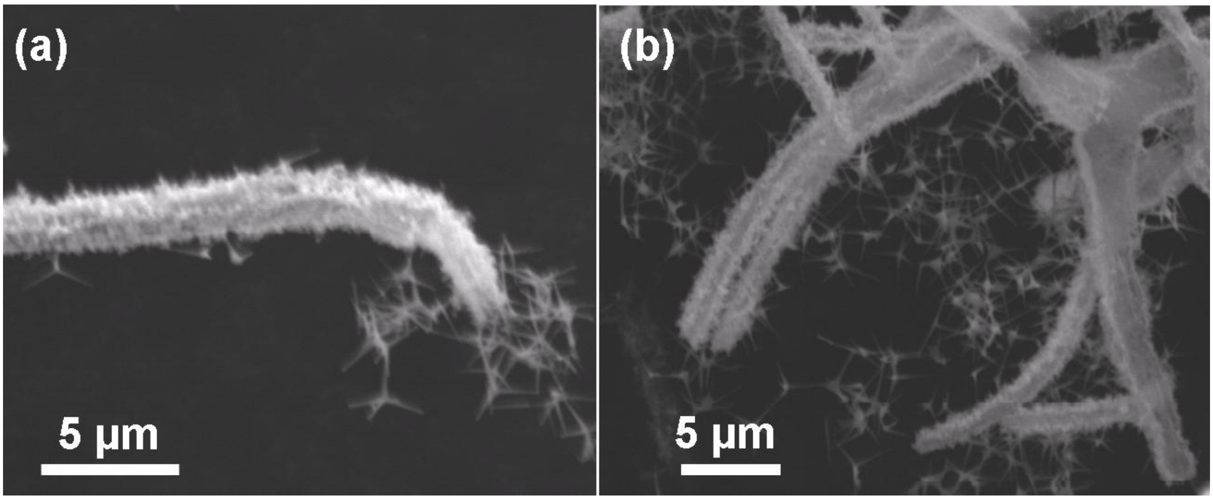

Figure S1. SEM images taken from ZnO/AG hybrid structures and detached ZnO micro-tetrapods, see text for details. The thickness of the ZnO nanocrystalline film deposited on Aerographite was 2 µm.

Figure S2

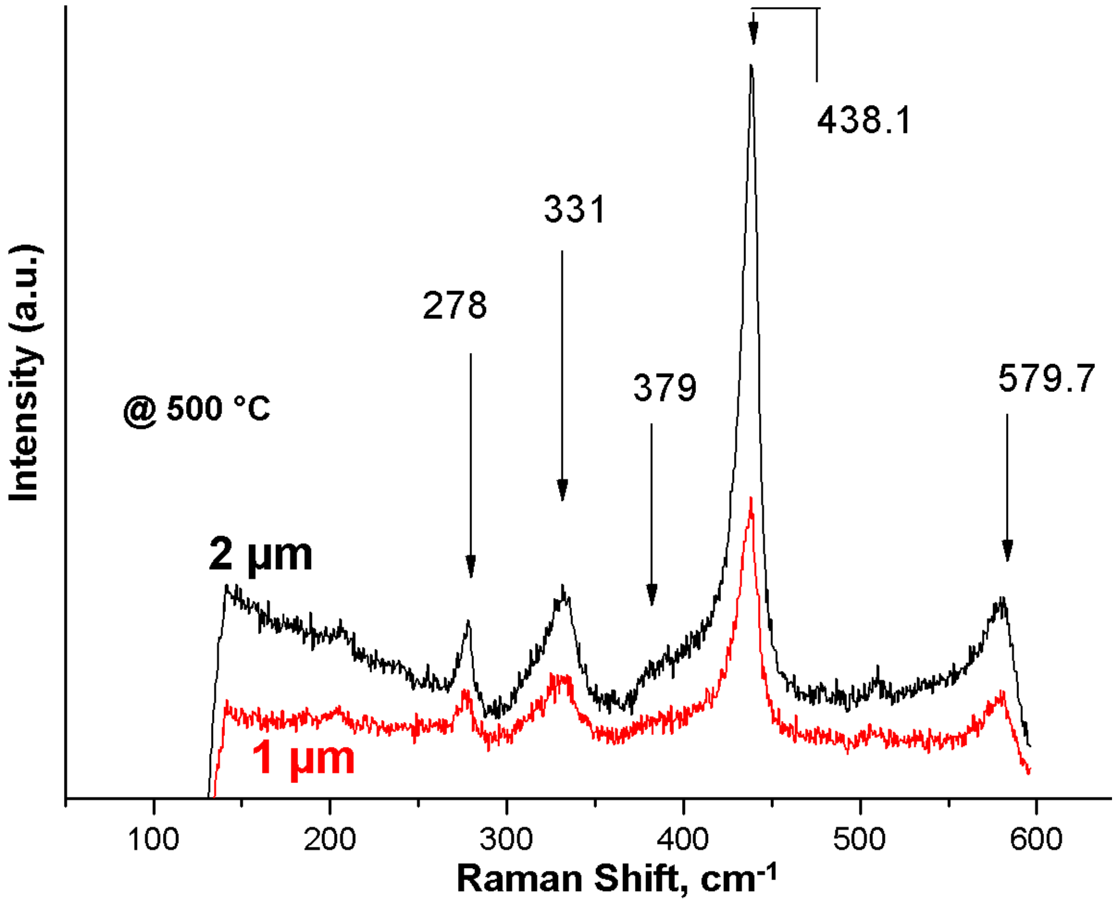

Figure S2: Micro-Raman scattering spectra taken from ZnO-Aerographite networks. With increase in the thickness of the deposited ZnO layers (red – 1 µm; black – 2 µm), the Raman peaks become more prominent demonstrating the enhanced growth of nanocrystalline ZnO with the increase in deposition time.

Figure S3

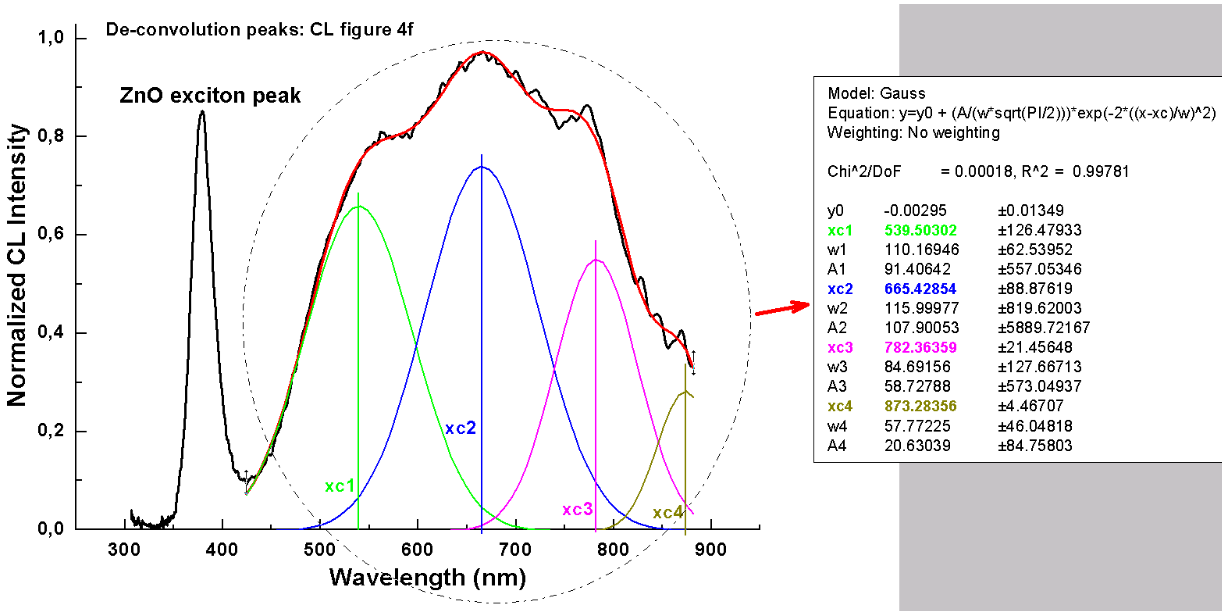

Figure S3: Deconvolution peaks corresponding to CL spectrum (figure 4f) for the highest amount of ZnO nano- and microstructures on Aerographite network. It can be clearly seen that there is significant luminescence in the infrared region which is most likely due to the contributions from intrinsic ZnO defects as well as carbon defects.

Figure S4

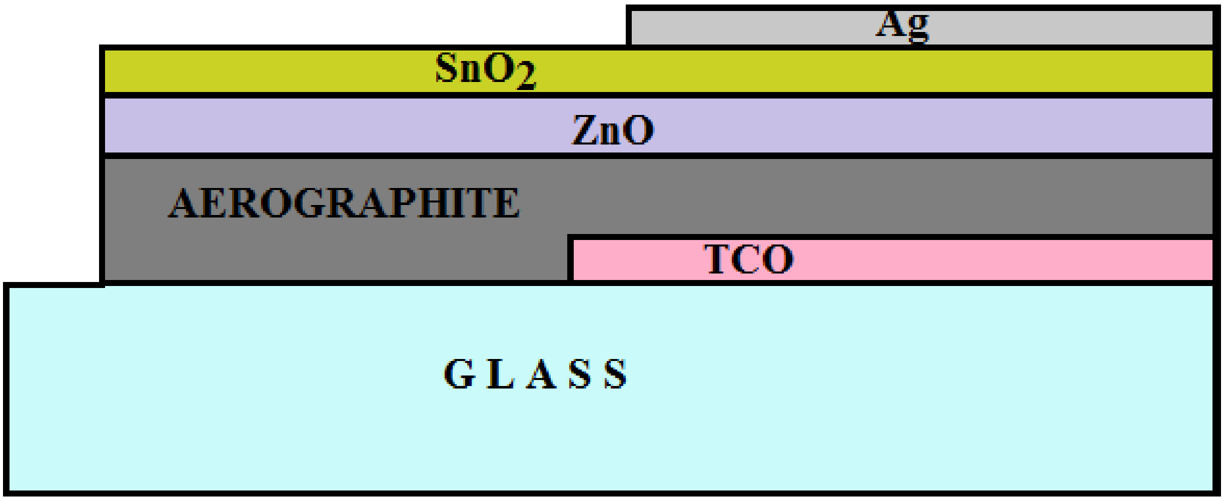

Figure S4. Schematic structure of TCO/aerographite/ZnO/SnO_2/Ag specimen.

Figure S5

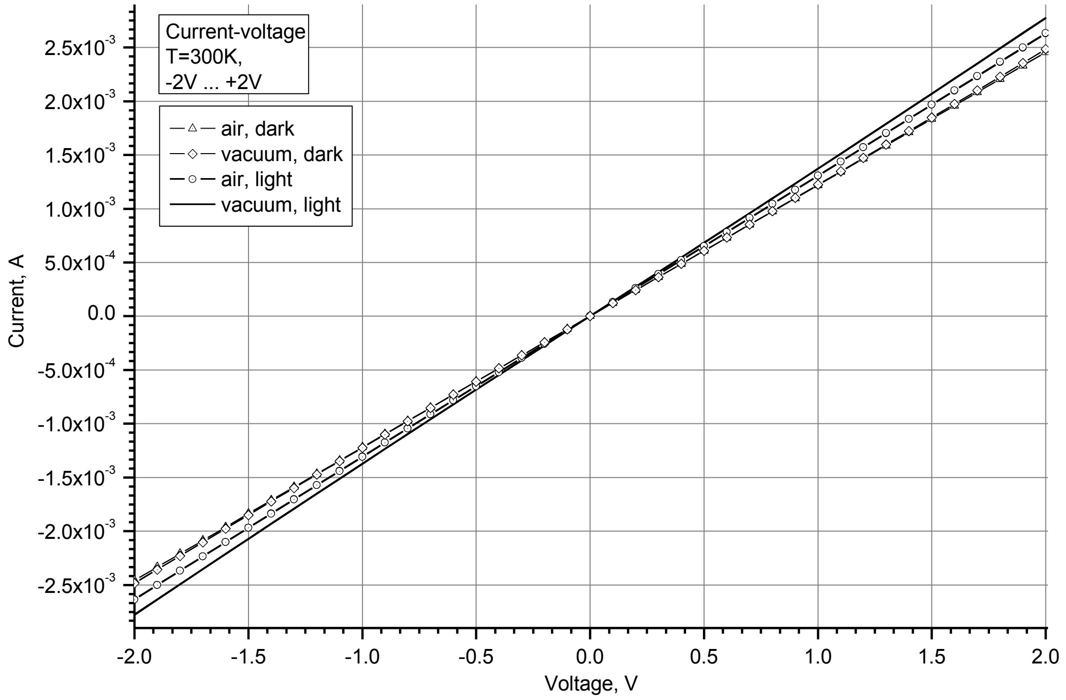

Figure S5. The volt-ampere characteristics of the ZnO/Aerographite hybrid structure.

Figure S6

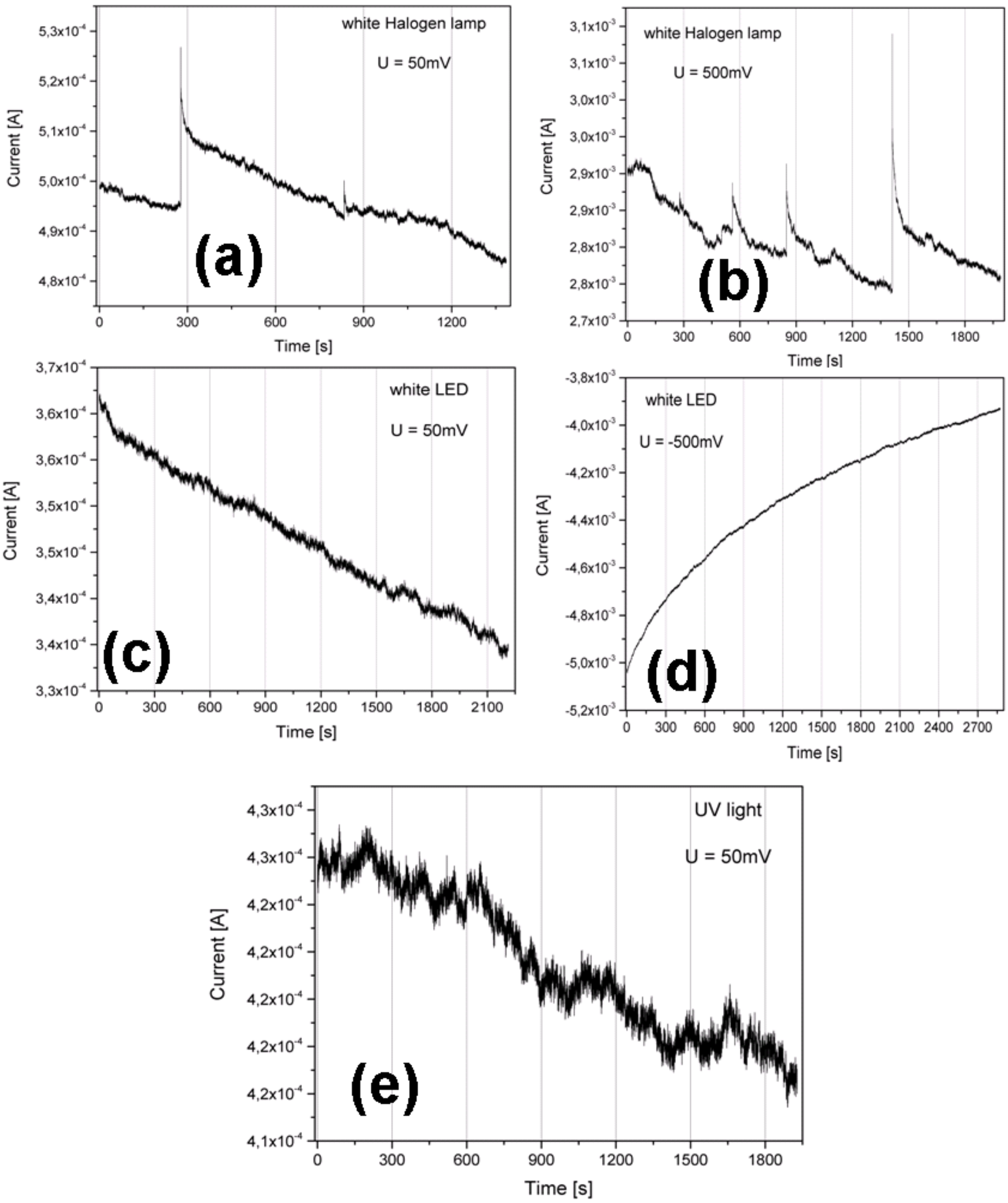

Figure S6. Photosensitivity response of pure Aerographite network in UV and visible regions of the spectrum (On/Off switching of the light source was performed in 5 min. intervals). The results of the characterization of pure Aerographite show that no dependence of the current on light was observed, giving evidence that the photosensitivity observed in AG-ZnO hybrid network (Figure 6) is due to the ZnO structures (a); (b) Measurement of current over time with a white halogen lamp as light source at two different voltages of 50 mV and 500 mV. The single peaks that occur during On/Off switching can be attributed to thermal excitations due to the hot halogen lamp (c); (d) Photosensitivity measurement with a white LED lamp at a voltage of 50 mV and -500 mV, respectively. (e) Photosensitivity measurement of Aerographite in UV light with an applied voltage of 50 mV.
